# 14-3-3γ Induces Oncogenic Transformation by Stimulating MAP Kinase and PI3K Signaling

**DOI:** 10.1371/journal.pone.0011433

**Published:** 2010-07-02

**Authors:** Vijayababu M. Radhakrishnan, Jesse D. Martinez

**Affiliations:** 1 Arizona Cancer Center, University of Arizona, Tucson, Arizona, United States of America; 2 Department of Cell Biology and Anatomy, Arizona Cancer Center, University of Arizona, Tucson, Arizona, United States of America; Roswell Park Cancer Institute, United States of America

## Abstract

The 14-3-3 proteins are a set of highly conserved scaffolding proteins that have been implicated in the regulation of a variety of important cellular processes such as the cell cycle, apoptosis and mitogenic signaling. Recent evidence indicates that the expression of some of the family members is elevated in human cancers suggesting that they may play a role in tumorigenesis. In the present study, the oncogenic potential of 14-3-3γ was shown by focus formation and tumor formation in SCID mice using 14-3-3γ transfected NIH3T3 mouse fibroblast cells. In contrast, 14-3-3σ, a putative tumor suppressor, inhibited NIH3T3 transformation by H-ras and c-myc. We also report that activation of both MAP kinase and PI3K signaling pathways are essential for transformation by 14-3-3γ. In addition, we found that 14-3-3γ interacts with phosphatidylinositol 3-kinase (PI3K) and TSC2 proteins indicating that it could stimulate PI3K signaling by acting at two points in the signaling pathway. Overall, our studies establish 14-3-3γ as an oncogene and implicate MAPK and PI3K signaling as important for 14-3-3γ induced transformation.

## Introduction

14-3-3 proteins are a family of highly conserved proteins that interact with and regulate a diverse array of cellular proteins. Seven family members have been identified in mammalian cells and each is designated by a Greek letter (β, γ, ε, η, σ, τ (θ) and ζ). Interaction between 14-3-3 proteins and their client proteins is mediated primarily, but not exclusively, through phosphoserine-binding via a consensus motif that can be phosphorylated by serine and threonine kinases [1]. As a consequence of this, 14-3-3 proteins are involved in a wide variety of signal regulated cellular processes [2] including receptor-mediated signal transduction [3], apoptosis [4], cell cycle progression [5] and checkpoint activation [6]. Many of the pathways regulated by 14-3-3 proteins become aberrantly activated during tumorigenesis. Not unexpectedly, there is mounting evidence that 14-3-3 proteins play a role in the development of human tumors [7]. This notion is most compellingly supported by studies which show that the expression of one family member, 14-3-3σ, is down regulated in breast cancers [8] and it also acts as a tumor suppressor [9]. On the other hand, 14-3-3ζ has been shown to cooperate with the Erb2 receptor to induce epithelial mesenchymal transformation in breast cancer suggesting that this family member is oncogenic [10]. Moreover, we and others have shown that 14-3-3 expression is aberrantly up regulated in several tumor types including lung cancer [11], [12], [13]. This suggests that abnormal expression of these proteins have a role in cancer; however, the mechanism by which these proteins promote neoplastic progression is not well understood.

Here we have focused our attention on 14-3-3γ because it is one of the several 14-3-3 proteins that is up regulated in non small cell lung cancer and also because it was shown previously that it can indirectly down regulate the transactivation of p53 tumor suppressor [14]. Using focus formation assays, we show that 14-3-3γ is an oncogene and that it functions by activating the MAPK and PI3K pathways. 14-3-3σ, in contrast, suppresses focus formation and inhibits activation of both of these signaling pathways.

## Materials and Methods

### Cell Culture and Transfection Conditions

NIH3T3 cell line was obtained from ATCC (American Type Tissue Culture) and MEF cells were provided by Dr. Thomas Doetschman, BIO5 Institute, University of Arizona, and these cells were routinely grown in Dulbecco's modified minimal essential medium (Cellgro, VA) supplemented with 2 mM L-glutamine, nonessential amino acids, 100 U of penicillin, 100 µg of streptomycin, and 6–10% fetal calf serum and maintained in a humidified atmosphere of 5% CO_2_.

### Plasmids

The T24-C3 (activated H-ras inserted into pBR322 plasmid) vector was obtained from Dr. Barbacid, NIH, MD, USA. pCMV6-c-myc and pCMV-14-3-3γ Flag vectors are laboratory stocks. The 14-3-3σ construct (Gene accession No. AFO29082) was made by PCR amplification of 14-3-3σ cDNA and sub-cloning into the pGEM Easy Vector (Promega, WI). A BamHI/EcoRI fragment of 14-3-3σ DNA was finally ligated into the pCMV-Flag-2b expression vector (Strategene, CA).

### Transformation assay

Transformation of NIH3T3 cells was performed according to the method as described earlier [15]. Briefly, early passages of NIH3T3 and MEF cells were plated in 30 mm, 6-well plates at a density of 1×10^5^cells/well. For transfection, 5–10 µg of each plasmid was used with LipoTAXI transfection agent (Strategene, CA). After 48 h, the transfected cells were trypsinized and plated onto 100 mm plates. Cells were kept in DMEM with 6% calf serum; the medium was changed every 3–4 days. After 21 days, cells were stained with 0.005% crystal violet solution and foci counted using a colony counter.

### Soft agar assay

Randomly selected transformed cell colonies were tested in triplicates for anchorage-independent growth in soft agar by previously described procedure [16]. To determine colony forming efficiency, 1×10^3^ cells were plated in 35 mm dishes. Fresh liquid medium (0.2 ml) was layered over the surface once a week to prevent desiccation. After three weeks, the colonies were stained with INT (2-(4-Iodophenyl)-3-(4-nitrophenyl)-5-phenyltetrazolium chloride, Sigma Aldrich, MO) and the number of large colonies (0.1 mm in diameter) with dense centers were counted.

### Tumorgenicity in SCID mice

SCID mice were obtained from the University of Arizona SCID resource. Mice were housed in micro isolator cages and provided with sterile food and water and treated following the conditions approved by the Institutional Animal Care and Use Committee for animal experiments, (Approval Number #09-076). To assess tumor formation in vivo, 14-3-3γ transformed cells (1×10^7^ cells per mouse) were implanted subcutaneously into the flanks of SCID mice. Tumor formation was assessed every 3–4 days for a total of 25 days.

### Raf-1 assay

Raf-1 immunoprecipitations and kinase assays were performed as described earlier [17]. Transfected NIH3T3 cells were grown for two days, and then starved overnight, PBS-washed and lysed with RIPA buffer and the lysate cleared by centrifugation at 10,000 g (10 min at 4°C). 200 µg of the protein lysate was added to 2 µg monoclonal anti-Raf-1 antibody (Cell Signaling Technology, MA) prebound to 30 µl of protein A/G-agarose beads (Pierce, IL) and incubated for 1 h at 4°C on a slow rotator. The Raf-1 immunocomplex was washed three times in ice-cold RIPA buffer, once in ice-cold washing buffer containing 10 mM HEPES (pH 7.4), 100 mM NaCl, 20 µg/ml aprotinin and 0.5% NP-40, and once in ice-cold reaction buffer containing 20 mM Tris (pH 7.4), 20 mM NaCl, 1 mM DTT and 10 mM MgCl_2_. Half of the immunocomplex was used for the kinase reaction and the other half for the Raf-1 immunoblot. The kinase reaction was performed in the presence of 20 mM ATP and 500 ng of recombinant MEK-1 substrate (Upstate, MA), in a total volume of 40 µl of reaction buffer at 30°C for 30 min with gentle agitation. Raf-1 immunocomplex was added to the reaction mixture containing kinase reaction buffer (25 mM HEPES, pH 7.4; 25 mM glycerol phosphate; 1 mM dithiothreitol; 10 mM manganese chloride; 100 mM ATP and 10 µCi of [γ-^32^P] ATP (Perkin Elmer, MA). The reaction was terminated by the addition of 40 µl 2×SDS sample buffer followed by boiling for 5 min. Samples were resolved by SDS-PAGE gel. Gels were fixed, dried and quantified using a Molecular Phosphorimager (Amersham Biosciences, NJ).

### ERK assay

ERK assay was performed as described earlier [18]. Briefly, transiently transfected NIH3T3 cells were starved overnight, washed with phosphate buffered saline and 200 µg protein samples were combined with ERK2 antibody (Santa cruz, CA) and incubated at 4°C for 4 h with gentle rocking, followed by an additional 1 h incubation with protein A/G (Pierce, IL). The pellets were then washed and resuspended with 50 µl kinase buffer supplemented with 200 mM ATP and 2 µg Elk-1 fusion protein (Cell Signalling, MA), and incubated at room temperature for 45 min. The reaction was stopped by adding lysis buffer and the proteins separated by SDS-10% PAGE. After immunoblotting with rabbit antiphospho- Elk-1 antibody (Cell Signalling, MA), the blot was stripped with anti-ERK2 (Rabbit polyclonal, Santa Cruz, CA) to confirm equal loading. Each experiment was repeated three times and quantified.

### PI3K assay

After transient transfection, cell-free lysates were prepared as described and PI3K assays performed by a previously described [19] method, in which cells were lysed in the presence of protease inhibitors. After normalization for protein content, the cleared lysates were incubated with p110-PI3K antibody (Santa Cruz, CA) for 2 h at 4°C, followed by addition of protein A/G-agarose beads (Pierce, IL) and an additional 1 h incubation. The immunoprecipitates were then washed three times, as described previously. The kinase reactions contained 20 mM Tris-HCl, pH 7.6, 75 mM NaCl, 10 mM MgCl_2_, 2.5 mM EGTA, 0.2 mg/ml phosphatidylinositol, 0.3 mg of phosphatidylserine, 20 mM ATP, 10 µCi of [γ-^32^P] ATP and were carried out at room temperature for 10 min and then stopped with 100 µl of 1 N HCl. Phospholipids were extracted twice, first with 200 µl of CHCl_3_∶MeOH (1∶1) and then with 160 µl of 1 N HCl∶MeOH (1∶1), after which the organic phase was dried and resuspended in 50 µl of CHCl_3_∶MeOH (1∶1). Phosphorylated products were resolved on oxalate impregnated Silica gel-60 plates (Merck, NJ) using CHCl_3_∶MeOH∶Acetone∶EtOH∶H_2_O (73∶48∶20∶20∶19) as solvent. Phosphatidylinositol 3-kinase activity was determined by autoradiography and quantified using a Phosphorimager (Amersham Biosciences, NJ).

### Immunoprecipitation

For co-immunoprecipitation experiments using lysates from cells stably expressing 14-3-3γ and 14-3-3σ proteins, 1 mg of lysate was mixed with 5 µg of anti-Flag antibody (Sigma Aldrich, MO), and incubated at 4°C overnight. Then, 30 µL of protein A/G beads (Pierce, IL) were added to each sample and shaken for 2 h. The beads were then washed three times with RIPA buffer and resuspended in 30 µl of 2×SDS-PAGE loading buffer and the samples were ran on a gel and immunoblotted with the antibodies noted in the results.

### GST-pull down and in vitro kinase assays

All 14-3-3 recombinant proteins were expressed in *BL21 E. coli* strain. GST-14-3-3 proteins were purified using glutathione-Sepharose 4B resin (Amersham Biosciences, NJ), according to the manufacturer's instructions. Purified proteins were tested for purity by gel electrophoresis and Coomassie blue staining. For in vitro kinase assays, the indicated amount of recombinant GST-14-3-3 protein was mixed with immunoprecipitates of Raf-1 and PI3K beads, incubated at 37°C for 2 h, and the kinase assay performed as described earlier. For binding assays, GST or GST-14-3-3 bound beads were incubated with precleared NIH3T3 cell lysates for 2 h at 4°C. Proteins bound to beads were washed three times with phosphate buffered saline and resuspended in 2×SDS-PAGE loading buffer. Samples were resolved by 10% SDS–PAGE and probed with antibodies to Raf-1, PI3K, TSC2 or GST.

## Results

### 14-3-3γ induces whereas 14-3-3σ suppresses transformation of NIH3T3 and MEF cells

**Figure 1:**
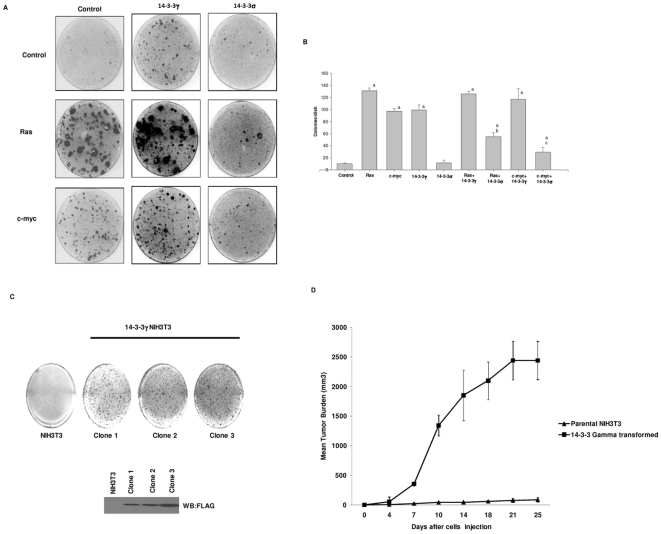
14-3-3γ, but not 14-3-3σ, induces focus formation in NIH3T3 cells. (A) H-ras, c-myc, 14-3-3γ and 14-3-3σ expression plasmids were transfected into NIH3T3 cells as described in the methods section, replated 48 h after transfection and maintained for an additional 21 days. Subsequently, the cells were stained with crystal violet and the number of foci quantified using an Oxford Optronix colony counter, UK. The experiment was repeated twice. A typical experiment is depicted. (B) The number of foci in panel A was determined and graphed. Bars depict the average number of foci per dish. (a, p<0.05 vs control; b, p<0.05 vs H-ras; c, p<0.05 vs c-myc, Student's t-test). (C) Several 14-3-3γ transformed foci from the experiment in panel A were collected and tested for growth in soft agar. Twenty one days after seeding, the colonies were stained. The growth of parental NIH3T3 cells is shown for comparison. (D) One of the clones from panel C was injected into eight SCID mice at 1×10^7^ cells per injection. Tumor volume was measured every 3–4 days for a total of 25 days. For comparison, NIH3T3 cells were injected into SCID mice as a control.

Studies have shown that several of the 14-3-3 genes may have oncogenic potential when over expressed in immortalized cells or in cancer-derived cell lines [10], [20]. To our knowledge, 14-3-3 proteins have not been tested for oncogenic activity using the focus formation assay to directly test for transforming ability. Here we have focused on the 14-3-3γ isoform because we had previously shown that elevated expression of this family member appeared to induce genetic instability [21]. To test for oncogenic activity we utilized the focus formation assay [22] whereby NIH3T3 or MEF cells were transfected with an expression vector that constitutively expressed the 14-3-3γ gene. For comparison, we also transfected cells with activated H-ras or c-myc expression vectors which are well recognized for their oncogenic activity. In addition to these, we also introduced 14-3-3σ, because this family member is reported to be a tumor suppressor [9]. As can be seen in figures 1A and 1B, introduction of 14-3-3γ into NIH3T3 cells resulted in the formation of foci that are similar in number and appearance to what was observed when c-myc was introduced into the cells, indicating that 14-3-3γ can function as a transforming oncogene. Moreover, the number of foci that occurred increased when 14-3-3γ was co-transfected with c-myc, suggesting 14-3-3γ could cooperate with this oncogene. Interestingly, H-ras co-transfection with 14-3-3γ did not result in more foci, indicating that 14-3-3γ does not act synergistically with H-ras, suggesting that these two oncogenes may transform through the same mechanism. As expected, 14-3-3σ was unable to induce focus formation in this assay, but instead could suppress focus formation when cotransfected with either H-ras or c-myc. The same results were observed in MEF cells (Figure S1).

To confirm the transforming ability of 14-3-3γ we utilized in vitro and in vivo assays to characterize the ability of 14-3-3γ transformed cells to grow in soft agar and form tumors in SCID mice, respectively. Several 14-3-3γ-induced foci from the experiments depicted in figure 1A were expanded and expression of 14-3-3γ confirmed by immunoblotting. The cells were then tested for anchorage independent growth on soft agar (Figure 1C). As can be seen, the 14-3-3γ transformed cells showed marked anchorage independent growth, when compared to the control. We next tested one of the expanded clones (clone 2) for ability to form tumors in SCID mice. The cells were implanted subcutaneously in SCID mice and the mice monitored for twenty five days after implantation (Figure 1D). 14-3-3γ transformed cells produced tumors that were first measurable at about day 7 and which continued to grow in size until termination of the experiment. No tumors were observed in mice injected with control cells. From these results, we conclude that 14-3-3γ can act as an oncogene.

**Figure 2:**
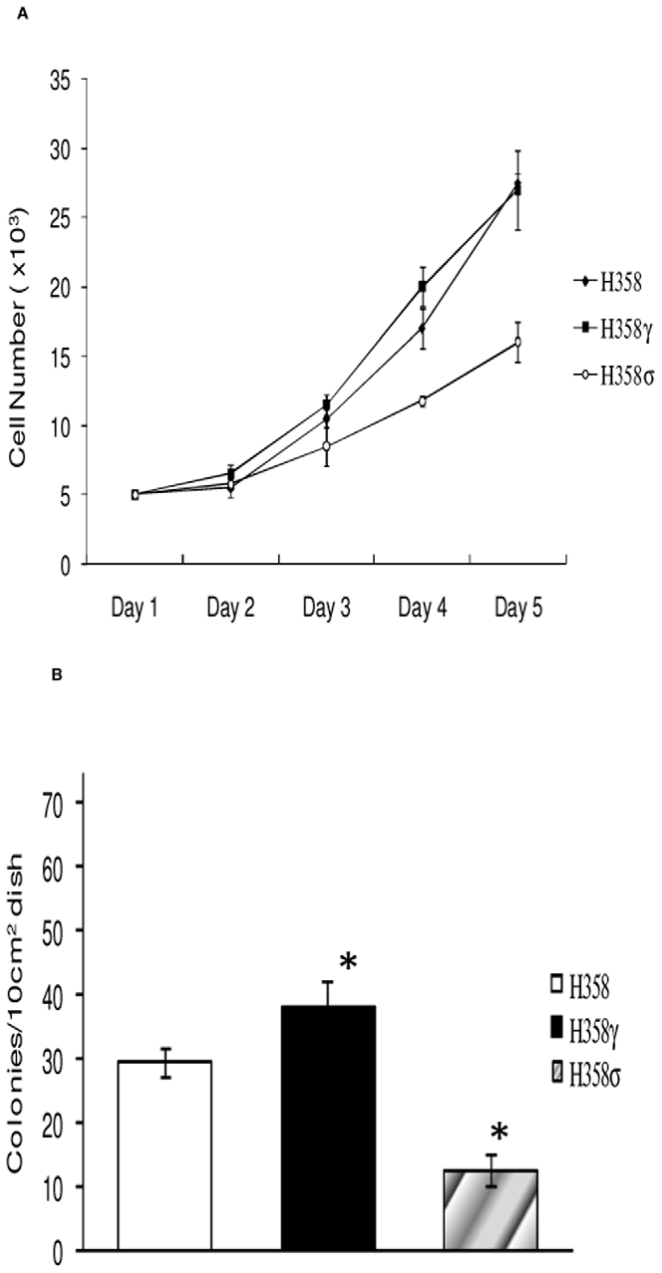
14-3-3σ, suppresses cell growth in a p53 independent manner. (A) H358 lung cancer cells stably transfected with 14-3-3γ or 14-3-3σ were seeded on 35 mm dishes at 1×10^3^ cells per plate and the cells cultured for 5 days. The cells in sample dishes were trypsinized and counted every 24 h. The results from four independent experiments are graphed. *, p<0.05 vs. control, Student's t-test. (B) The same cell lines as in A were tested for growth in soft agar as described in figure 1. The average results of three independent experiments are graphed. *, p<0.05 vs control, Student's t-test.

Previous studies have shown that exogenous expression of 14-3-3β can alter the growth characteristics of cells [20]. Hence, to further explore 14-3-3γ's transforming ability, we expressed 14-3-3γ in H358 lung cancer cell lines. For comparison, we also utilized 14-3-3σ and found that while 14-3-3γ did not increase proliferation rates it did enhance anchorage independent growth (Figures 2A and 2B). In contrast, 14-3-3σ suppressed both proliferation and anchorage independent growth of H358 cells. Overall, our results indicate that 14-3-3γ may function through the same mechanism as H-ras in causing transformation and that facilitating anchorage independent growth may be an important activity of 14-3-3γ oncogenic function.

### MAPK and PI3K pathways are required for 14-3-3γ transformation

**Figure 3:**
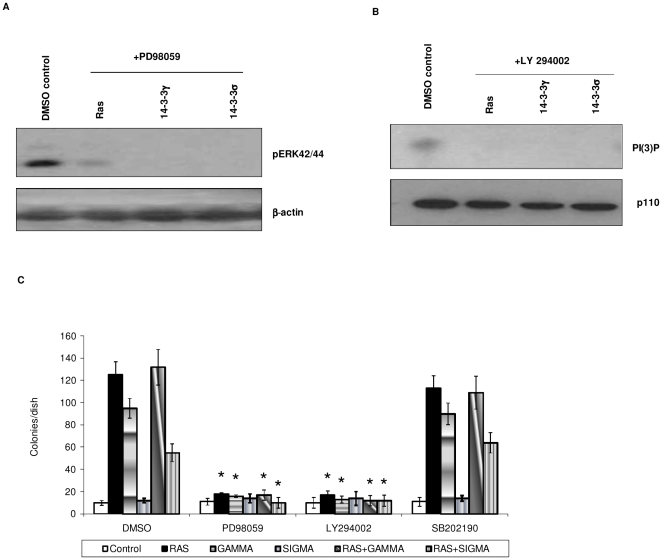
Transformation by 14-3-3γ requires active MAPK and PI3Kinase pathways. NIH3T3 cells were transfected with H-ras, 14-3-3γ, or 14-3-3σ plasmids and incubated for 48 hours in DMEM medium containing either 15 µM PD98059 or 5 µM LY294002. DMEM supplemented with DMSO served as a control. After 48 h, the cells were harvested and extracts prepared and examined for the presence of phosphorylated ERK42/44 by immunoblotting (A) or tested for PI3K activity (B) as described in the methods section. These experiments were repeated twice. A representative figure is shown. (C) NIH3T3 focus formation assays were conducted as in figure 1 using either H-ras, 14-3-3γ, 14-3-3σ, or a combination of H-ras plus 14-3-3γ or 14-3-3σ and in media with or without 15 µM PD98059, 5 µM LY294002 or 1 µM SB202190. The assays were maintained in an incubator at 37°C and 5% CO_2_ for 21 days and the colonies stained and counted. The bars represent the average number of colonies per 100 mm dish from two independent experiments. Error bars depict standard deviation. (*, p<0.05, Student's t-test, compared with control assay conducted in the absence of inhibitor).

The lack of cooperation between 14-3-3γ and H-ras suggested that 14-3-3γ may utilize the same or a parallel pathway as H-ras in causing transformation. Consistent with this, it has been shown that 14-3-3 proteins interact with and may activate raf [23], the MAP kinase that is stimulated by H-ras and which is essential for H-ras-mediated transformation [24]. Recent evidence suggests that PI3K also plays an important role in H-ras transformation [25]. Hence, to test for involvement of MAPK and PI3K pathways in 14-3-3γ induced transformation, we used PD98059 and LY294002, which inhibit MAPK and PI3K signaling respectively, in the transformation assay. For comparison we also suppressed p38 MAP kinase signaling using SB203580. We first determined drug concentrations that did not affect NIH3T3 cell viability (data not shown) but did suppress signaling activity (Figures 3A and 3B). After transfection, the cells were incubated with these inhibitors and focus formation was measured after 21 days. We found that both PD98059 and LY294002 suppressed transformation induced by H-ras (positive control) and 14-3-3γ whereas the p38 inhibitor, SB203580 showed no effect on focus formation (Figure 3C). Collectively, these results suggested that activation of both MAPK and PI3K signaling is required for transformation by 14-3-3γ.

### 14-3-3γ induces activation of MAPK signaling in vivo

**Figure 4:**
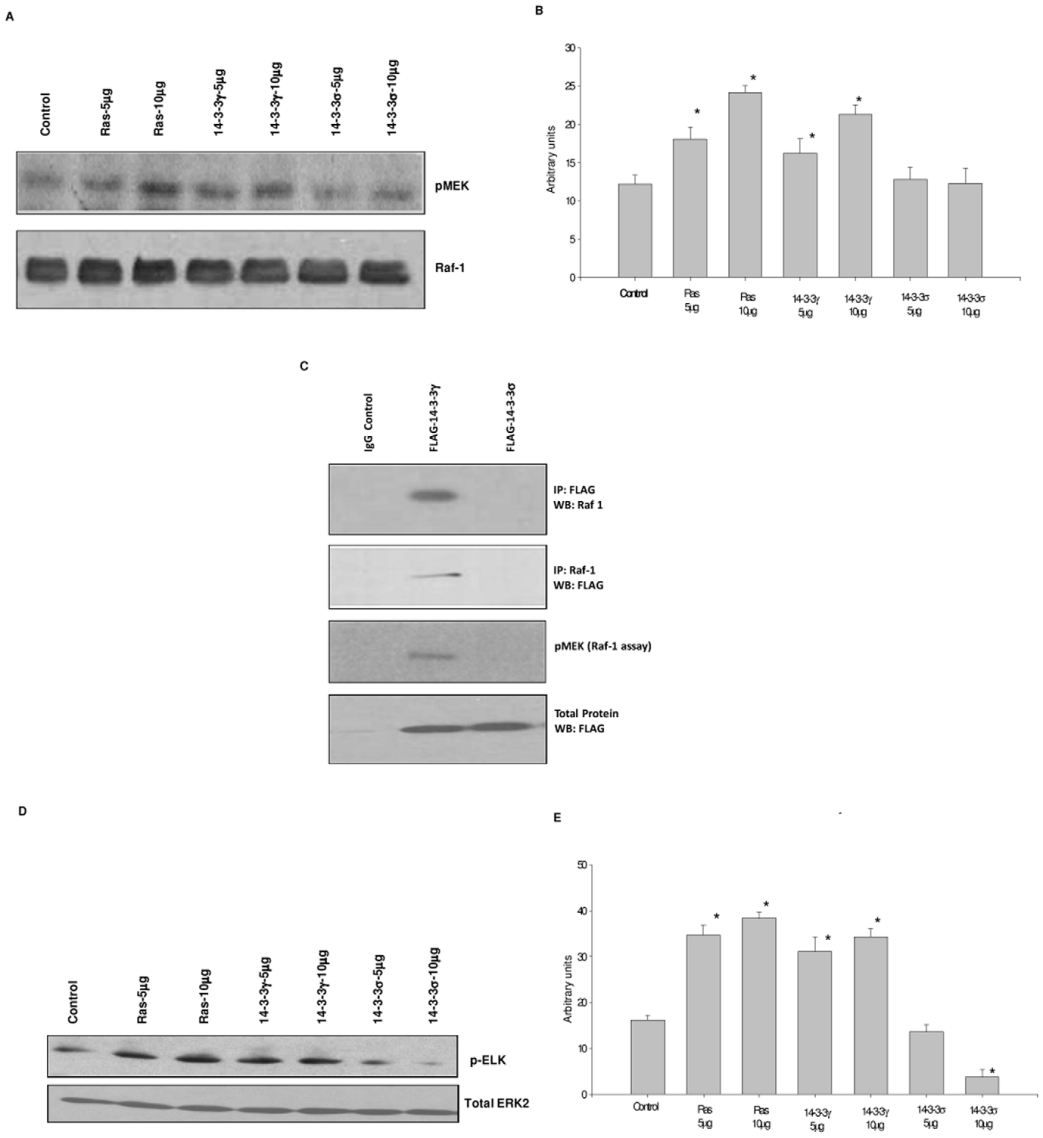
14-3-3γ activates MAPK signaling. (A) Increasing quantities of H-ras, 14-3-3γ, 14-3-3σ, expression plasmids or vector only (control) were transiently transfected into NIH3T3 cells. Forty eight hours after transfection, the cells were serum starved overnight, harvested, and cell extracts examined for Raf-1 activity by incubating with recombinant MEK protein in the presence of [γ-^32^P] ATP. Total raf-1 protein in the extracts was determined by immunoblotting (bottom panel). The experiment was repeated three times. A typical experiment is depicted. (B) The amount of radioactivity in the pMEK bands in panel A was quantified. Bars in the graph are the average for the three experiments. Error bars depict standard deviation (*, p<0.05 vs. vector control). (C) Extracts were prepared from NIH3T3 cells stably transfected with FLAG-14-3-3γ or FLAG-14-3-3σ. 14-3-3 proteins were immunoprecipitated with anti-FLAG antibody and the precipitated proteins tested for the presence of raf-1 by immunoblotting (top panel). Raf-1 protein was immunoprecipitated from the same extracts and tested for the presence of 14-3-3 proteins using the anti-FLAG antibody (second panel). The extracts were also tested for raf-1 kinase activity as described above (third panel). Total 14-3-3 protein was detected in the extracts by immunoblotting using the anti-FLAG antibody (bottom panel). (D) NIH3T3 cells were transiently transfected with increasing quantities of H-ras, 14-3-3γ, or 14-3-3σ expression plasmids as indicated. Forty eight hours later the cells were harvested, extracts prepared and ERK2 activity determined by incubating with recombinant Elk-1 protein and phosphorylated ELK detected by immunoblotting using an anti-phosphorylated-ELK antibody. Total ERK2 protein was detected by immunoblotting using an anti-ERK2 antibody (bottom panel). The experiment was repeated three times. A typical experiment is depicted. (E) The density of the bands in panel D was quantified using Image J (NIH, MD). Bars in the graph are the average of the three experiments. Error bars depict standard deviation (*, p<0.05 vs. control, Student's t-test).

We next tested for activation of raf-1 signaling by 14-3-3γ. NIH3T3 cells were transfected with H-ras, 14-3-3γ, or 14-3-3σ and then raf-1 tested for its ability to phosphorylate MEK (Figures 4A and 4B). As expected, transfecting H-ras into the cells resulted in an increased raf-1 activity as measured by its ability to phosphorylate MEK. Similarly, transfecting 14-3-3γ into the cells also increased raf-1 activity. In contrast, there was no change in raf-1 activity in cells transfected with 14-3-3σ. To confirm these results we immunoprecipitated 14-3-3γ and 14-3-3σ from transiently transfected cells and then tested for binding with raf-1 and for raf's ability to phosphorylate MEK (Figure 4C). We found that raf-1 co-immunoprecipitated with 14-3-3γ, but not 14-3-3σ and that the raf-1 associated with 14-3-3γ could phosphorylate MEK. We further examined the effects of 14-3-3 on MAPK signaling by testing for their effects on ERK activity (Figures 4D and 4E). Transfection of either H-ras or 14-3-3γ led to a dose dependent increase in ERK activity. However, introduction of 14-3-3σ caused a dose dependent decrease in ERK activity. Our results indicate that 14-3-3γ can stimulate MAP kinase signaling in vivo, whereas 14-3-3σ suppresses this signaling pathway.

### 14-3-3γ activates PI3K signaling, whereas 14-3-3σ inhibits it

**Figure 5:**
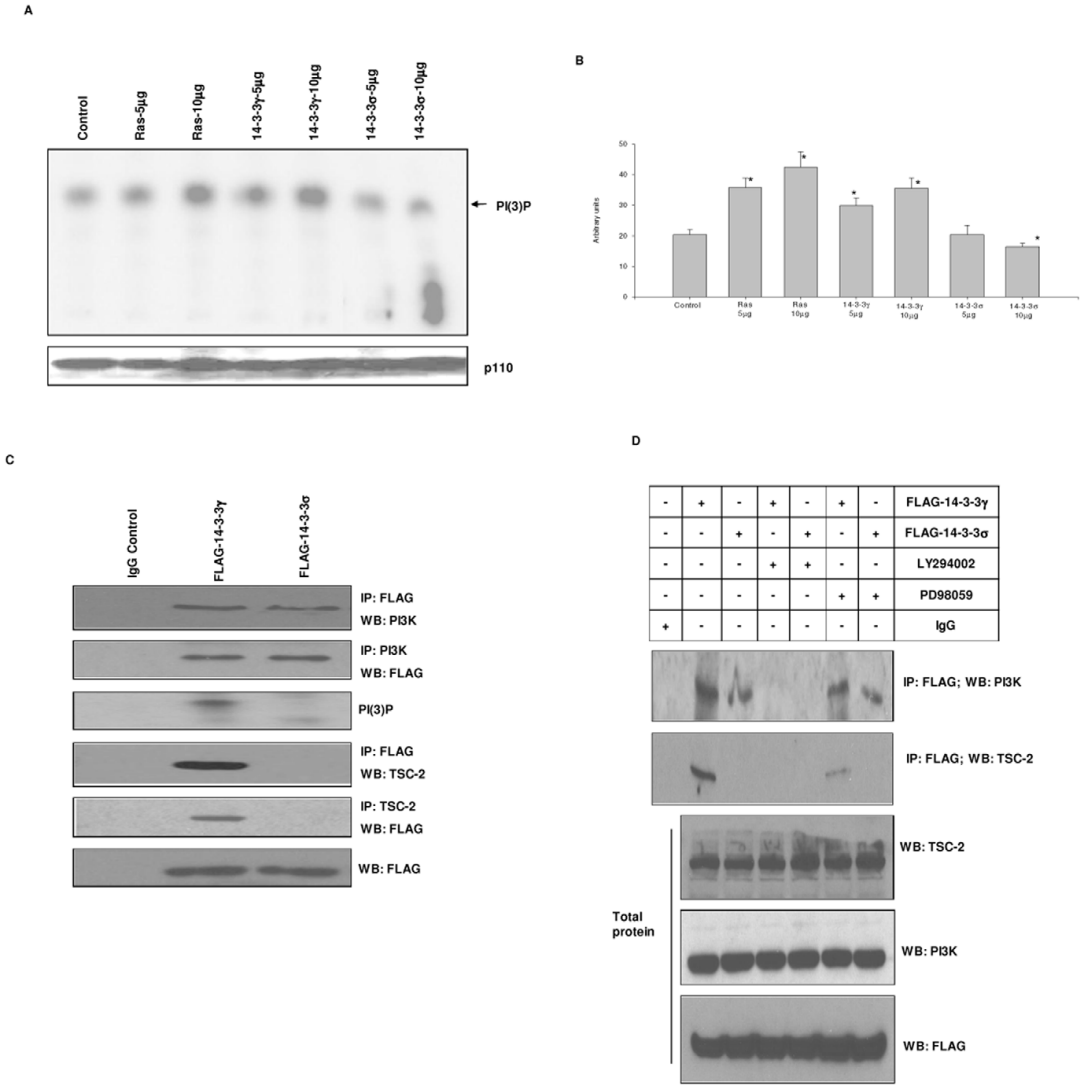
14-3-3γ stimulates PI3K activity. (A) Increasing quantities of H-ras, 14-3-3γ and 14-3-3σ expression plasmids were transiently transfected into NIH3T3 cells. Forty eight hours later the cells were serum starved overnight and then the cells harvested. The PI3K-p110 catalytic subunit was immunoprecipated and catalytic activity measured by incubating with phosphatidyl inositol and [γ-^32^P] ATP. Radiolabled phosphatidyl inositol was detected using thin layer chromatography (Top panel). Parallel aliquots of the same precipitates were resolved by SDS-PAGE and immunoblotted with anti-p110 antibody (bottom panel). The experiment was repeated three times. A typical result is depicted. (B) Radioactivity in the PI (3)-P spots from panel A was quantified. Bars in the graph are the average of the three experiments. Error bars depict standard deviation (*, p<0.05 vs. control, Student's t-test). (C) Extracts from NIH3T3 cells stably expressing FLAG-14-3-3γ or FLAG-14-3-3σ were prepared. 14-3-3 proteins were immunoprecipitated using an anti-FLAG antibody and probed for the presence of PI-3 kinase using an anti-p110 antibody (top panel). PI-3 kinase p110 subunit was immunoprecipitated and the precipitated proteins probed for the presence of 14-3-3 using an anti-FLAG antibody (second panel). PI3K activity was detected in the FLAG antibody immunoprecipitates by incubating with phosphatidyl inositol as described previously (third panel from the top). 14-3-3 proteins were immunoprecipitated with the anti-FLAG antibody and the immunoprecipitated proteins probed for the presence of TSC2 by immunoblotting with an anti-TSC2 antibody (fourth panel from the top). TSC2 protein was immunoprecipitated from the extracts and the precipitated proteins probed for the presence of 14-3-3 proteins by immunoblotting with the anti-FLAG antibody. Total 14-3-3 protein in the extracts was detected by immunoblotting with the anti-FLAG antibody. An IgG control is shown for comparison. (D) 14-3-3γ and 14-3-3σ stably expressing cells were treated with either PD98059 or LY294002 as indicated. 14-3-3 proteins were immunoprecipitated with an anti-FLAG antibody and the immunoprecipitated proteins probed for PI-3 kinase by immunoblotting with an anti-p110 antibody (top panel). In a parallel experiment, the FLAG antibody immunoprecipitated proteins were probed for the presence of TSC2 (second panel). Finally, the extracts were probed for total level of TSC2 (third panel), PI-3 kinase p110 subunit (fourth panel from the top), and 14-3-3 protein (bottom panel) by immunoblotting with the appropriate antibody. Proteins immunoprecipitated using an IgG control are shown for comparison.

In the above experiments we found that the LY294002 inhibitor could suppress focus formation by 14-3-3γ suggesting that activation of PI3K signaling was important for 14-3-3γ-mediated oncogenic activity. This prompted us to test for PI3K activation in cells that were transiently transfected with 14-3-3γ or 14-3-3σ (Figures 5A and 5B). Both H-ras and 14-3-3γ were potent activators of PI3K, whereas 14-3-3σ suppressed activity. This is consistent with the concept that 14-3-3γ can stimulate PI3K signaling.

It has been shown that other 14-3-3 proteins can directly bind components of the PI3K signaling pathway including p110-PI3K and TSC2 [26], [27]. Hence we tested for an interaction between these two proteins and 14-3-3γ and 14-3-3σ. To explore this, we created cell lines that stably expressed FLAG-tagged 14-3-3γ and 14-3-3σ and tested for an interaction between the 14-3-3 proteins and p110-PI3K and TSC2 using immunoprecipitation (Figure 5C). Immunoprecipitation of the 14-3-3 proteins followed by immunoblotting for p110-PI3K revealed that p110 could bind to both 14-3-3γ and 14-3-3σ and this was confirmed by the reciprocal immunoprecipitation experiment. Importantly, only the p110-PI3K bound with 14-3-3γ showed catalytic activity suggesting that 14-3-3γ could activate this enzyme. In contrast, when the immunoprecipitated 14-3-3 proteins were tested for the presence of TSC2, TSC2 was found in complex with 14-3-3γ only and not with 14-3-3σ. Taken together these results indicate that 14-3-3γ and 14-3-3σ have distinctly different effects on the PI3K signaling pathway.

The above experiments suggested that the interactions between 14-3-3γ and p110-PI3K and TSC2 may be important in the stimulation of PI3K signaling. Moreover, since pharmacological inhibition of PI3K and MAPK pathways suppressed 14-3-3γ-mediated focus formation we reasoned that the PD98059/LY294002 inhibitors may affect the protein complexes formed between 14-3-3γ and proteins of the PI3K pathway. NIH3T3 cells that stably expressed either 14-3-3γ or 14-3-3σ were treated with LY294002 and the 14-3-3 proteins immunoprecipitated using the FLAG antibody. For comparison, cells were also incubated with PD98059 in a separate set of experiments (Figure 5D). As can be seen, incubating cells with LY294002 abolished the interaction between 14-3-3γ and both p110-PI3K and TSC2. It also eliminated coimmunoprecipitation of p110-PI3K with 14-3-3σ. PD98059 did not eliminate the interaction between the 14-3-3 and p110-PI3K, but it did reduce the interaction with TSC2. Taken together, our results indicate that 14-3-3γ can interact with at least two components of the PI3K signaling pathway and that this interaction is suppressed when signaling activity through this pathway is inhibited.

### 14-3-3γ can stimulate PI3K activity, but not raf-1 activity, in vitro

**Figure 6:**
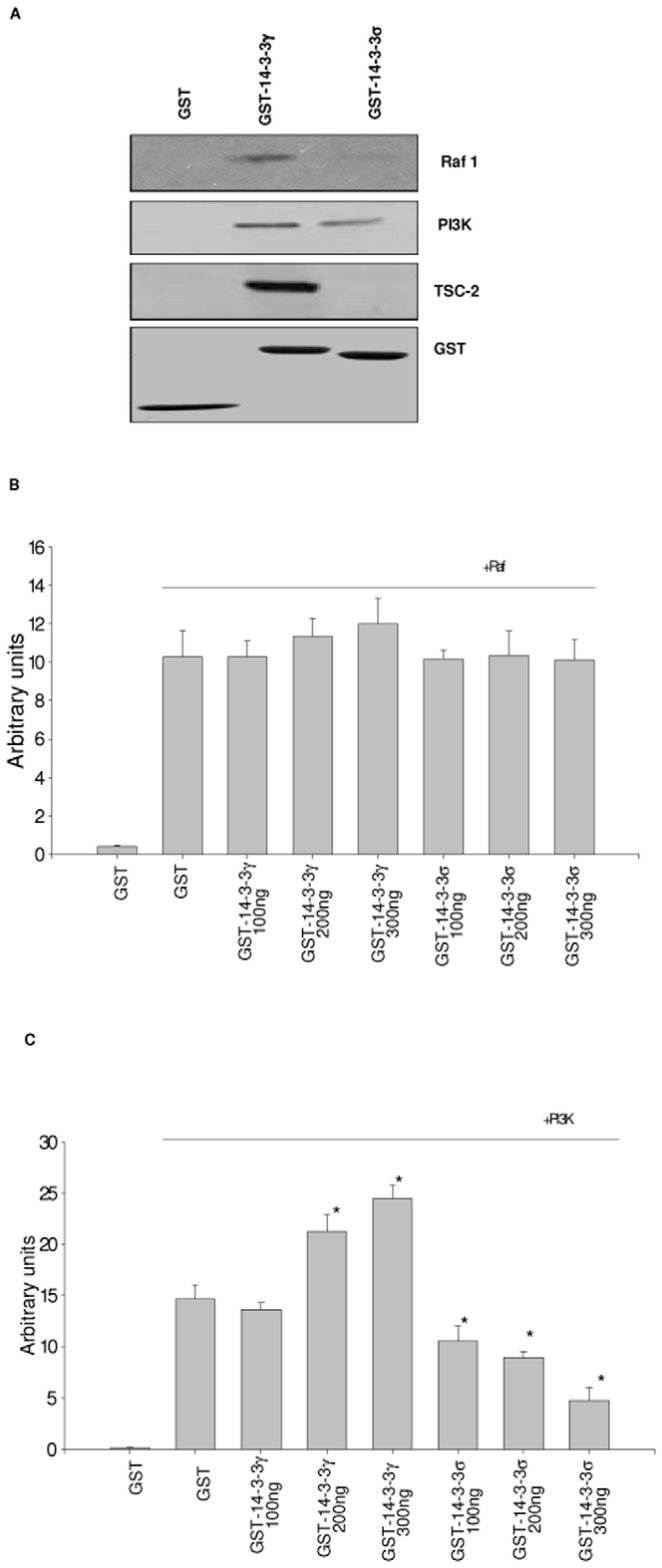
14-3-3γ and 14-3-3σ interact with MAPK and PI3K components in vitro. (A) Glutathione-sepharose beads were loaded with purified recombinant GST-14-3-3 proteins and then incubated with cleared NIH3T3 cell lysates. The beads were washed and then the recovered proteins probed for the presence of Raf-1, PI3K p110 subunit, and TSC2 by immunoblotting with the appropriate antibody. A representative blot was stripped and reprobed with anti-GST antibody (bottom panel). (B) Raf-1 assay was performed on Raf-1 immunoprecipitate in the presence of GST or GST-14-3-3 proteins using recombinant MEK protein as a substrate. The reaction mixture was subjected to SDS-PAGE and analyzed by autoradiography. The levels of GST-fusion proteins used in the kinase assays were examined by Coomassie blue staining. (C) PI3K assay was performed using p110 immunoprecipitates in the presence of GST or GST-14-3-3 fusion proteins using 0.2 mg PI as substrate. The products were resolved on TLC and quantified by phopshoImager. (*, p<0.05 vs. PI3K beads alone, Student's t-test).

One of the several mechanisms that 14-3-3 proteins are thought to act is by altering the conformation of enzymes that they interact with causing their activation [28]. We showed that 14-3-3γ could stimulate MAPK and PI3K signaling which prompted us to test whether 14-3-3γ and 14-3-3σ could alter the activity of the signaling kinases that they interacted with. To accomplish this, we generated 14-3-3γ and 14-3-3σ GST fusion proteins and purified them from *E.coli* and confirmed that our recombinant proteins could interact with raf-1, p110-PI3K, and TSC2 (Figure 6A). We next immunopurified raf-1 from NIH3T3 cells and then added increasing quantities of purified GST-14-3-3 proteins (Figure 6B). Our tests for changes in basal activity showed that 14-3-3γ had no effect on raf-1 activity, indicating that direct physical interaction between 14-3-3γ and raf-1 did not modify basal raf-1 activity. As expected, 14-3-3σ had no effect on raf-1 activity since we showed previously that it did not interact with this MAP kinase. We also tested for the in vitro effects of adding purified 14-3-3γ and 14-3-3σ to immunopurified p110-PI3K (Figure 6C). In contrast to what we found with raf-1, 14-3-3γ could stimulate p110-PI3K activity in a dose dependent manner, whereas 14-3-3σ suppressed its activity. These results indicate that 14-3-3 proteins can modify the activity of p110-PI3K in vitro and that 14-3-3γ and 14-3-3σ have opposing effects on this kinase.

## Discussion

The primary observation we have made is that 14-3-3γ functions as an oncogene and that it's transforming activity is mediated through activation of MAP kinase and PI3K signaling. We previously reported that 14-3-3γ is overexpressed in lung cancer [13] implying that this 14-3-3 family member has oncogenic potential. In this study, we found that the overexpression of 14-3-3γ in NIH3T3 or MEF cells resulted in focus formation. These transformed cells exhibited anchorage-independent growth in soft agar and tumor formation in SCID mice, both hallmarks of the transformed phenotype. Importantly, we show that overexpression of 14-3-3γ leads to constitutive activation of the MAPK and PI3K pathways. Similarly, Takihara, et al. [20] reported that increased MAPK signaling activity was observed in the 14-3-3β transformed cells. Since increased activation of MAPK and PI3K pathways can result in malignant transformation [29], [30], we suppressed ERK/PI3K activation with PD98059/LY294002 inhibitors and found that inhibition of either signaling pathway decreased foci formation by 14-3-3γ. This suggests that the concerted action of both pathways is essential for transformation by this oncogene.

Our co-immunoprecipitation experiments showed that 14-3-3γ interacts with Raf-1 and this was also confirmed in vitro GST binding assays. Interestingly, Raf-1 kinase activity could be stimulated by 14-3-3γ in vivo but not in vitro where we utilized purified protein. Activation of Raf-1 is a complex event that requires multiple accessory proteins that were not present in our in vitro experiment. Hence, 14-3-3γ alone could not activate Raf-1 in vitro. This is consistent with previous study carried out by [31], where they showed that Raf-1 could not be activated by purified GST-14-3-3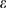 recombinant protein. However, overexpression of 14-3-3γ did lead to increased Raf-1 activity in vivo and this could be explained by several ways. 14-3-3γ may stabilize the conformation required for substrate recognition and catalytic activity or may mediate the interaction of Raf-1 with other downstream signaling molecules. Another possibility is that 14-3-3γ could facilitate the intracellular trafficking involved in the translocation of Raf-1 where it becomes activated.

Studies have shown that 14-3-3 proteins play an important role in the PI3K-Akt signaling pathway by interacting with TSC2 protein [32], [33], [34]. This interaction is phosphorylation-dependent, requiring TSC2 phosphorylation on S939 and S981 by AKT [27]. This prompted us to study the interaction of 14-3-3 and TSC2 proteins by immunoprecipitation and we found that TSC2 binds to 14-3-3γ protein both in vivo and in vitro. TSC2 is a critical mediator of both MAPK and PI3K pathways [35], [36], [37], [38], hence we tested whether the interaction between 14-3-3γ and TSC2 was affected by the presence of PD98059/LY294002 inhibitors. We observed that both these inhibitors strongly reduced the interaction of 14-3-3γ with TSC2. This is in conjunction with the suppression of focus formation in the presence of PD98059/LY294002 inhibitors, suggested that transformation by 14-3-3γ requires interaction with TSC2 and activation of mTOR.

14-3-3σ is down regulated by promoter methylation or ubiquitin-mediated degradation [39], [40] and acts as a tumor suppressor. Our study confirmed this in co-transfection studies with H-ras and c-myc, wherein 14-3-3σ markedly reduced the transformation induced by H-ras in NIH3T3 cells. We also detected decreased ERK2 and PI3K activity in 14-3-3σ expressing cells. Previously, Yang et al. [41] showed that 14-3-3σ binds to AKT and inactivates its function and, since both AKT and PI3K lie in the same pathway; it is possible that 14-3-3σ might block both these enzyme activities simultaneously. Hence 14-3-3σ may function as a tumor suppressor by inhibiting the same pathways that are activated by 14-3-3γ. Therefore; 14-3-3γ and 14-3-3σ have opposing biological activities.

In conclusion, the results presented in this report show that activation of MAPK and PI3K pathways are essential for 14-3-3γ induced transformation. Furthermore, suppression of these pathways by 14-3-3σ could be one of the mechanisms of its tumor suppressor function. A critical question, however, is how these proteins function differently even though they share more than 85% homology in their structure. We are currently investigating the transforming ability and tumor suppressor function of these proteins.

## Supporting Information

Figure S1 Transformation assay in MEF cells. H-ras and 14-3-3 expression plasmids were transfected into MEF cells as described in “Materials and Methods.” Transformed foci were stained with crystal violet.
(0.19 MB TIF)
