# Supplementary figures and images for: 14-3-3γ Induces Oncogenic Transformation by Stimulating MAP Kinase and PI3K Signaling
Source: PLoS One. 2010 Jul 2;5(7):e11433. doi: 10.1371/journal.pone.0011433 (PMC2900177; doi:10.1371/journal.pone.0011433)

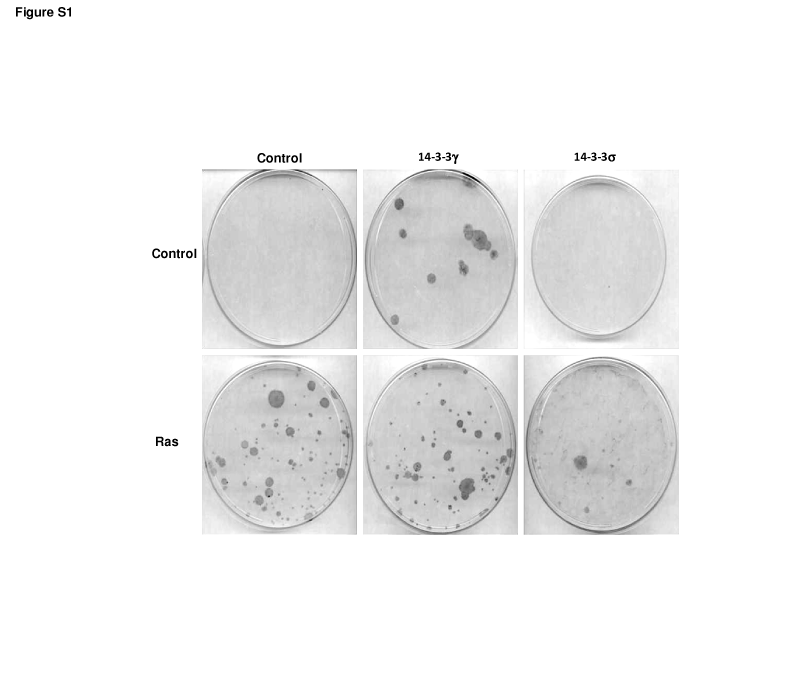

Supplement: Figure S1 — Transformation assay in MEF cells. H-ras and 14-3-3 expression plasmids were transfected into MEF cells as described in “Materials and Methods.” Transformed foci were stained with crystal violet. (0.19 MB TIF) [file pone.0011433.s001.tif]
